# Subjective irregular sleep is associated with metabolic syndrome: A cross-sectional study

**DOI:** 10.1016/j.pmedr.2022.101844

**Published:** 2022-05-23

**Authors:** Yasuhiro Ogura, Teruhide Koyama, Etsuko Ozaki, Chie Omichi, Ritei Uehara

**Affiliations:** a Department of Epidemiology for Community Health and Medicine, Kyoto Prefectural University of Medicine, Kyoto 602-8566, Japan; b Department of Psychiatry, Shiga University of Medical Science, Shiga 520-2192, Japan; c Department of Hygiene and Public Health, Osaka Medical and Pharmaceutical University, Takatsuki, 569-8686, Japan

**Keywords:** Metabolic syndrome, Sleep irregularity, Sleep duration, Bedtime

## Abstract

Several studies have been reported that sleep duration and circadian rhythms are associated with metabolic syndrome (MetS). However, there are few studies of a relationship between sleep and MetS based on subjective evaluation of sleep regularity. The aim of this study is to examine the relationship between subjective sleep irregularity and metabolic syndrome.

This cross-sectional study included 3,880 participants (1,383 males, 2,497 females) from 2013 to 2017, and we use a self-administered questionnaire to acquire information about sleep (sleep regularity, duration and bedtime). Odds ratios (ORs) and 95% confidence intervals (CIs) were calculated using logistic regression analyses to evaluate the associations between sleep regularity and the prevalence of MetS.

The irregularity of sleep was significantly associated with MetS (OR 1.231, 95% CI 1.101–1.375) adjusted for age, sex, METs, sleep duration, bedtime, drinking and smoking statuses, and a history of using sleeping pills. We examined the interaction of MetS with sleep regularity and sleep duration/bedtime, stratified by multiplying the two groups of sleep regularity/irregularity and the three groups of sleep duration/bedtime. Each group of sleep duration/bedtime showed no relationship in the sleep regularity group with MetS, but a significant relationship in the sleep irregularity group. Leptin was significantly elevated in the irregular sleep group regardless of sleep duration and bedtime.

Although many studies have shown a link between sleep and MetS especially in terms of sleep duration, this study showed that irregular sleep is more strongly associated with MetS than sleep duration or bedtime.

## 1 Introduction

Metabolic syndrome (MetS) has been associated with an increased risk of several cancers (Esposito et al., 2012), cardiovascular diseases (Mottillo et al., 2010), type 2 diabetes (Morrison et al., 2008), and mortality (Mottillo et al., 2010). Inappropriate diet, lack of exercise, excessive alcohol consumption, and work-related psychological factors have been reported as causes of MetS (Eckel et al., 2005, Grundy, 2008, Watanabe et al., 2018), along with irregular sleep. One longitudinal study showed that sleep problems are mediators between occupational stress and metabolic syndrome (Garbarino and Magnavita, 2019). Several studies have analyzed the relationship between sleep duration and MetS and results show that short sleep duration is significantly associated with MetS (Iftikhar et al., 2015, Xi et al., 2014). Some studies have shown that both short and long sleep times, such as the U-shape, are associated with MetS (Smiley et al., 2019, Xie et al., 2021). Along with the quantity of sleep, the circadian rhythm is an equally important component of sleep (Zimmet et al., 2019). Sleep and circadian rhythms are known to regulate and control the daily patterns of physiology in humans and are important for maintaining healthy metabolic functions. Daily patterns of energy expenditure (Markwald et al., 2013), hormones, and lipids involved in energy metabolism (for example, glucose, insulin, fatty acids, triglycerides, and leptin) are regulated by sleep and circadian rhythms (Nguyen and Wright, 2010, Poggiogalle et al., 2018, Spiegel et al., 2011).

In modern society, the misalignment of circadian rhythms caused by jet lag due to artificial light at night, shift work, and travel to different time zones have become common, and there is a growing concern that sleep irregularities disrupt the metabolic system. A previous *meta*-analysis concluded that there is currently sufficient evidence to support the conclusion that shift work is associated with MetS (Khosravipour et al., 2021); however, most studies use objective data to estimate sleep regularity. The advantage of subjective assessment is that it does not require a device and can be used by anyone, anywhere, anytime, and in large numbers of subjects. This simplicity will also be useful for future epidemiological studies. However, few studies have investigated the relationship between sleep and MetS based on the subjective evaluation of sleep regularity. In this study, we evaluated the relationship between subjective sleep irregularity and MetS.

## 2 Material and methods

### 2.1 Study population and design

We previously performed a cohort study, known as the J-MICC study (Japan Multi-Institutional Collaborative Cohort Study) (Takeuchi et al., 2020). This cross-sectional study included individuals who were enrolled in the J-MICC study’s second survey in the Kyoto area from 2013 to 2017 (Haraguchi et al., 2019). Among the 3,913 participants, 33 were excluded because their data had missing values. Thus, the total sample comprised 3,880 participants (1,383 males, 2,497 females). The study was approved by the Institutional Ethics Committee of the Kyoto Prefectural University of Medicine (approval number: ERB-E-36-8, 2013) and was conducted in accordance with the principles of the Declaration of Helsinki. All participants provided written informed consent prior to participation.

### 2.2 Data collection and measurements

We used a standardized self-administered questionnaire to acquire information about each participant’s sleeping time (hour/day), smoking and drinking statuses (in three categories: never, former, and current), daily physical activity times, and venous blood sampling. We asked about sleep regularity as follows: “Do you have regular hours to sleep and wake up?” (regular or irregular). The average sleep duration was categorized based on the tertile value: <6h/day, T1; 6–<7h/day, T2; and ≥7 h/day, T3. The average bedtime was categorized on the basis of the tertile value: <23:00, 23:00 to <24:00, and ≥24:00, T1 to T3 respectively. Physical activity was determined using a format similar to that of the International Physical Activity Questionnaire (Craig et al., 2003). Physical activity was assessed in terms of metabolic equivalents (METs), as previously reported (Hara et al., 2012). In brief, METs-hours per day of activity was estimated by multiplying the reported daily time spent in each activity by the relevant MET intensity.

Waist circumference (WC) was measured at the umbilical level with minimal respiration in a standing position. The body mass index was calculated as the weight divided by the square of height (kg/m^2^). Anamnesis and medication history were assessed using self-administered questionnaires. Hypertension was defined as a systolic/diastolic blood pressure ≥140/90 mmHg or the current use of medication for hypertension. Dyslipidemia was defined as low-density lipoprotein cholesterol levels ≥140 mg/dL, high-density lipoprotein cholesterol (HDL-C) levels <40 mg/dL, or the current use of medication for dyslipidemia. Diabetes was defined as a glycated hemoglobin (HbA1c) level ≥6.5%, blood sugar (BS) levels ≥126, or current use of medication for diabetes. The Japanese criteria for MetS were defined as follows: a male with WC ≥85 cm and a female with WC ≥90 cm in addition to two or more of the following: lipid abnormality, high triglyceride levels (≥150 mg/dL), HDL-C levels <40 mg/dL, or the use of lipid-modifying drugs; elevated blood pressure: systolic blood pressure ≥130 mmHg, diastolic blood pressure ≥85 mmHg, or the use of antihypertensive drugs; and elevated blood glucose levels: HbA1c levels ≥5.6%, BS levels ≥100, or the use of drugs for diabetes (Yamagishi and Iso, 2017). Leptin measurements were limited (n = 1,948).

### 2.3 Statistical analysis

The participants were divided into two groups according to sex. Continuous variables are expressed as means, and categorical data are expressed as sums and percentages. Intergroup comparisons were performed using one-way analysis of variance for continuous variables and chi-squared tests for categorical variables. Odds ratios (ORs) and 95% confidence intervals (CIs) were calculated using logistic regression analyses to evaluate the prevalence of MetS. We used three predictor variables as sleep factors: sleep regularity, sleep duration and bedtime. In Table 2, sleep regularity, sleep duration, and bedtime were used simultaneously as predictor variables. In Table 3, Table 4, the two groups in terms of sleep regularity/irregularity and the three groups (T1-T3) in terms of sleep duration or bedtime were stratified by multiplying each predictor variable to six groups. Covariates included the age, sex, METs, drinking and smoking statuses, and the current use of medication for sleeping pills. In Table 2, Model 1 was adjusted for age and sex. Model 2 was adjusted for age, sex, METs, drinking and smoking statuses. Model 3 was adjusted for age, sex, METs, drinking and smoking statuses, and the current use of medication for sleeping pills. Statistical significance was set at *p* < 0.05. All data were analyzed using the SPSS version 25.

**Table 1:** Characteristics of participants according to sleep regularity.

		Regular sleep		Irregular sleep		p-value
All	Sex (male), n [%]	1263	35.6	120	35.9	0.905
	Age (years), mean [SD]	57.7	9.9	55.5	10.3	<0.001
	BMI (kg/m^2^), mean [SD]	22.2	3.2	23.3	3.9	<0.001
	Waist Circumference (cm), mean [SD]	80.6	9.1	83.5	10.9	<0.001
	Systolic blood pressure (mmHg), mean [SD]	129	19.3	130	19.7	0.486
	Diastolic blood pressure (mmHg), mean [SD]	79.2	11.3	80.2	12.0	0.148
	Total cholesterol (mg/dL), mean [SD]	216	35.3	218	41.3	0.484
	Triglyceride (mg/dL), mean [SD]	99.1	67.9	116	104	0.004
	HDL-cholesterol (mg/dL), mean [SD]	70.6	17.4	67.1	16.8	<0.001
	LDL-cholesterol (mg/dL), mean [SD]	125	30.8	128	32.2	0.141
	Glucose (mg/dL), mean [SD]	90.5	14.8	90.9	13.6	0.665
	Hemoglobin A1C (%), mean [SD]	5.6	0.5	5.6	0.4	0.797
	METs (h/day), mean [SD]	14.5	10.4	14.6	10.9	0.923
	Sleep duration (h/day), interquatile range 25–75% [min–max]	6.0–7.0	2.5–11.0	5.0–6.0	3.0–9.0	<0.001
	Sleep duration, n [%]					
	T1 (<6h)	701	19.8	138	41.3	<0.001
	T2 (6 h to < 7 h)	1417	40.0	128	38.3	
	T3 (≥7h)	1428	40.3	68	20.4	
	Bed time, interquatile range 25–75% [min–max]	23:00–24:00	18:00–31:00	23:30–25:30	14:00–31:00	<0.001
	Bed time, n [%]					
	T1 (<23:00)	1456	41.1	73	21.9	<0.001
	T2 (23:00 to < 24:00)	1321	37.3	75	22.5	
	T3 (≥24:00)	769	21.7	186	55.7	
	Smokers, n [%]					
	Current	311	8.8	41	12.3	0.099
	Former	836	23.6	78	23.4	
	Never	2399	67.7	215	64.4	
	Current drinkers, n [%]					
	Current	2095	59.1	196	58.7	0.825
	Former	57	1.6	4	1.2	
	Never	1394	39.3	134	40.1	
	Hypertension, n [%]	1365	38.5	137	9.1	0.378
	Diabetes mellitus, n [%]	64	1.8	8	2.4	0.398
	Dyslipidemia, n [%]	1591	44.9	142	42.5	0.421
	Metabolic syndrome, n [%]	440	12.4	62	18.6	0.002
	Medication for sleeping pill, n [%]	191	5.4	31	9.3	0.006
	Leptin, mean (n) [SD]	11.3 (n = 1793)	8.3	16.6 (n = 155)	13.8	<0.001

Male	Age (years), mean [SD]	58.8	9.9	55.3	10.6	0.001
	BMI (kg/m^2^), mean [SD]	23.5	3.0	23.9	3.3	0.188
	Waist Circumference (cm), mean [SD]	84.7	8.2	86.3	9.5	0.085
	Systolic blood pressure (mmHg), mean [SD]	136	18.3	134	17.8	0.389
	Diastolic blood pressure (mmHg), mean [SD]	83.4	10.8	82.4	11.5	0.342
	Total cholesterol (mg/dL), mean [SD]	205	31.8	206	35.1	0.895
	Triglyceride (mg/dL), mean [SD]	121	85.2	144	109	0.031
	HDL-cholesterol (mg/dL), mean [SD]	61.9	15.8	60.2	14.2	0.202
	LDL-cholesterol (mg/dL), mean [SD]	122	29.3	121	31.4	0.879
	Glucose (mg/dL), mean [SD]	95.0	17.5	94.0	16.9	0.541
	Hemoglobin A1C (%), mean [SD]	5.6	0.6	5.6	0.4	0.470
	METs (h/day), mean [SD]	13.9	10.8	14.7	11.2	0.463
	Sleep duration (h/day), interquatile range 25–75% [min–max]	6:00–7:00	2:30–10:00	5:00–6:00	4:00–8:00	<0.001
	Sleep duration, n [%]					
	T1 (<6h)	205	16.2	42	35.0	<0.001
	T2 (6 h to < 7 h)	465	36.8	52	43.3	
	T3 (≥7h)	593	47.0	26	21.7	
	Bed time, interquatile range 25–75% [min–max]	23:00–24:00	18:00–31:00	23:00–25:00	14:00–29:30	0.002
	Bed time, n [%]					
	T1 (<23:00)	601	47.6	39	32.5	<0.001
	T2 (23:00 to < 24:00)	419	33.2	22	18.3	
	T3 (≥24:00)	243	19.2	59	49.2	
	Smokers, n [%]					
	Current	226	17.9	23	19.2	0.373
	Former	588	46.6	48	40.0	
	Never	449	35.6	49	40.8	
	Current drinkers, n [%]					
	Current	962	76.2	88	73.3	0.367
	Former	28	2.2	1	0.8	
	Never	273	21.6	31	25.8	
	Hypertension, n [%]	669	53.0	63	52.5	0.924
	Diabetes mellitus, n [%]	43	3.4	7	5.8	0.194
	Dyslipidemia, n [%]	563	44.6	48	40.0	0.387
	Metabolic syndrome, n [%]	338	26.8	38	31.7	0.283
	Medication for sleeping pill, n [%]	60	4.8	12	10.0	0.028
	Leptin, mean (n) [SD]	7.2 (n = 622)	5.4	7.2 (n = 50)	4.1	0.951

Female	Age (years), mean [SD]	57.0	9.8	55.7	10.0	0.061
	BMI (kg/m^2^), mean [SD]	21.4	3.0	22.9	4.2	<0.001
	Waist Circumference (cm), mean [SD]	78.3	8.7	81.9	11.3	<0.001
	Systolic blood pressure (mmHg), mean [SD]	126	18.9	128	20.4	0.169
	Diastolic blood pressure (mmHg), mean [SD]	76.9	10.9	79.0	12.2	0.016
	Total cholesterol (mg/dL), mean [SD]	223	35.6	225	42.9	0.430
	Triglyceride (mg/dL), mean [SD]	86.8	52.2	100	97.7	0.052
	HDL-cholesterol (mg/dL), mean [SD]	75.4	16.4	71.0	16.9	<0.001
	LDL-cholesterol (mg/dL), mean [SD]	128	31.5	132	32.0	0.050
	Glucose (mg/dL), mean [SD]	88.1	12.4	89.1	11.1	0.191
	Hemoglobin A1C (%), mean [SD]	5.5	0.4	5.6	0.4	0.330
	METs (h/day), mean [SD]	14.9	10.2	14.6	10.8	0.658
	Sleep duration (h/day), interquatile range 25–75% [min–max]	6:00–7:00	3:00–11:00	5:00–6:00	3:00–9:00	<0.001
	Sleep duration, n [%]					
	T1 (<6h)	496	21.7	96	44.9	<0.001
	T2 (6 h to < 7 h)	952	41.7	76	35.5	
	T3 (≥7h)	835	36.6	42	19.6	
	Bed time, interquatile range 25–75% [min–max]	23:00–24:00	18:00–31:00	24:00–26:00	21:00–31:00	<0.001
	Bed time, n [%]					
	T1 (<23:00)	855	37.5	34	15.9	<0.001
	T2 (23:00 to < 24:00)	902	39.5	53	24.8	
	T3 (≥24:00)	526	23.0	127	59.3	
	Smokers, n [%]					
	Current	85	3.7	18	8.4	0.001
	Former	248	10.9	30	14.0	
	Never	1950	85.4	166	77.6	
	Current drinkers, n [%]					
	Current	1133	49.6	108	50.5	0.955
	Former	29	1.3	3	1.4	
	Never	1121	49.1	103	48.1	
	Hypertension, n [%]	696	30.5	74	34.6	0.216
	Diabetes mellitus, n [%]	21	0.9	1	0.5	1.000
	Dyslipidemia, n [%]	1028	45.0	94	43.9	0.774
	Metabolic syndrome, n [%]	102	4.5	24	11.2	<0.001
	Medication for sleeping pill, n [%]	131	5.7	19	8.9	0.071
	Leptin, mean (n) [SD]	13.5 (n = 1171)	8.8	21.1 (n = 105)	14.6	<0.001

**Table 2:** The relationship between metabolic syndrome and the irregular sleep, bedtime, and sleep duration.

		Model 1			Model 2			Model 3		
		OR	95% CI	p-value	OR	95% CI	p-value	OR	95% CI	p-value
All	Irregular sleep	1.068	1.018–1.120	<0.001	1.230	1.101–1.375	<0.001	1.231	1.101–1.375	<0.001
	Bedtime T1	ref			ref			ref		
	Bedtime T2	0.987	0.773–1.260	0.916	1.066	0.838–1.356	0.603	1.066	0.838–1.356	0.604
	Bedtime T3	0.975	0.696–1.366	0.884	1.107	0.827–1.481	0.494	1.107	0.827–1.481	0.495
	Sleep duration T1	ref			ref			ref		
	Sleep duration T2	1.207	0.910–1.600	0.192	1.206	0.908–1.604	0.196	1.206	0.907–1.605	0.198
	Sleep duration T3	0.931	0.689–1.257	0.641	0.921	0.680–1.248	0.596	0.921	0.679–1.249	0.595

Male	Irregular sleep	1.032	0.968–1.101	0.328	1.135	0.983–1.310	0.084	1.135	0.983–1.310	0.085
	Bedtime T1	ref			ref			ref		
	Bedtime T2	0.975	0.729–1.305	0.867	1.017	0.765–1.354	0.906	1.017	0.764–1.354	0.906
	Bedtime T3	0.858	0.567–1.299	0.470	0.907	0.635–1.297	0.593	0.907	0.635–1.297	0.593
	Sleep duration T1	ref			ref			ref		
	Sleep duration T2	1.220	0.860–1.732	0.296	1.209	0.849–1.721	0.293	1.208	0.847–1.723	0.296
	Sleep duration T3	0.819	0.569–1.180	0.285	0.810	0.560–1.171	0.263	0.810	0.559–1.173	0.265

Female	Irregular sleep	1.105	1.033–1.183	0.004	1.365	1.154–1.615	<0.001	1.365	1.153–1.614	<0.001
	Bedtime T1	ref			ref			ref		
	Bedtime T2	1.135	0.709–1.816	0.598	1.254	0.789–1.993	0.339	1.255	0.789–1.995	0.337
	Bedtime T3	1.370	0.758–2.477	0.297	1.652	0.982–2.777	0.058	1.655	0.983–2.785	0.058
	Sleep duration T1	ref			ref			ref		
	Sleep duration T2	1.226	0.757–1.983	0.598	1.248	1.768–2.028	0.370	1.251	0.769–2.034	0.367
	Sleep duration T3	1.268	0.749–2.146	0.376	1.255	0.735–2.144	0.406	1.258	0.735–2.152	0.402
OR, odds ratio; CI, confidence interval										
Model 1 was adjusted for age and sex										
Model 2 was adjusted for age, sex, METs, drinking and smoking statuses										
Model 3 was adjusted for age, sex, METs, drinking and smoking statuses, and the current use of medication for sleeping pills

**Table 3:** The interaction of metabolic syndrome with sleep regularity and sleep duration.

		Number	Number of MetS	MetS (%)	OR	95% CI	p-value	Leptin, mean (n)	[SD]
All	Regular sleep × Sleep duration T1	701	81	10.1%	ref			11.5 (3 4 9)	7.7
	Regular sleep × Sleep duration T2	1417	184	13.0%	1.246	0.909–1.707	0.171	11.7 (7 2 6)	8.7
	Regular sleep × Sleep duration T3	1428	185	13.0%	0.927	0.667–1.288	0.651	10.9 (7 1 8)	8.2
	Irregular sleep × Sleep duration T1	138	24	17.4%	1.992	1.150–3.449	0.014	17.7 (68)	15.4
	Irregular sleep × Sleep duration T2	128	24	18.8%	1.997	1.149–3.472	0.014	14.0 (57)	11.5
	Irregular sleep × Sleep duration T3	68	14	20.6%	2.035	1.008–4.110	0.048	18.9 (30)	13.9

Male	Regular sleep × Sleep duration T1	601	164	27.3%	ref			7.3 (85)	4.6
	Regular sleep × Sleep duration T2	419	113	27.0%	1.234	0.838–1.816	0.287	7.3 (2 3 9)	6.1
	Regular sleep × Sleep duration T3	243	61	25.1%	0.846	0.570–1.255	0.405	7.1 (2 9 8)	5.0
	Irregular sleep × Sleep duration T1	39	16	41.0%	1.711	0.814–3.599	0.157	7.5 (17)	3.7
	Irregular sleep × Sleep duration T2	22	8	36.4%	1.927	0.980–3.788	0.057	6.0 (24)	3.6
	Irregular sleep × Sleep duration T3	59	14	23.7%	0.843	0.310–2.295	0.739	9.6 (9)	5.2

Female	Regular sleep × Sleep duration T1	855	31	3.6%	ref			12.8 (2 6 4)	8.1
	Regular sleep × Sleep duration T2	902	41	4.5%	1.287	0.741–2.236	0.371	13.9 (4 8 7)	8.9
	Regular sleep × Sleep duration T3	526	30	5.7%	1.123	0.612–2.061	0.707	13.5 (4 2 0)	9.0
	Irregular sleep × Sleep duration T1	34	6	17.6%	2.322	1.036–5.207	0.041	21.1 (51)	16.3
	Irregular sleep × Sleep duration T2	53	5	9.4%	2.142	0.822–5.580	0.119	19.7 (33)	11.8
	Irregular sleep × Sleep duration T3	127	13	10.2%	5.354	2.130–13.46	<0.001	22.9 (21)	14.6
OR, odds ratio; CI, confidence interval									
Adjusted for age, sex, METs, bedtime(T1-T3), drinking and smoking statuses, and the current use of medication for sleeping pills

**Table 4:** The interaction of metabolic syndrome with sleep regularity and bedtime.

		Number	Number of MetS	MetS (%)	OR	95% CI	p-value	Leptin, mean (n)	[SD]
All	Regular sleep × Bedtime T1	1456	195	13.4%	ref			10.4 (7 4 2)	8.0
	Regular sleep × Bedtime T2	1321	154	11.7%	1.091	0.850–1.402	0.494	11.5 (6 8 8)	8.3
	Regular sleep × Bedtime T3	769	91	11.8%	1.224	0.896–1.671	0.204	12.9 (3 6 3)	8.9
	Irregular sleep × Bedtime T1	73	22	30.1%	2.703	1.504–4.859	0.001	13.9 (34)	15.2
	Irregular sleep × Bedtime T2	75	13	17.3%	2.207	1.117–4.362	0.023	17.5 (31)	14.2
	Irregular sleep × Bedtime T3	186	27	14.5%	1.713	1.049–2.796	0.031	17.2 (90)	13.2

Male	Regular sleep × Bedtime T1	601	164	27.3%	ref			7.0 (2 9 8)	5.5
	Regular sleep × Bedtime T2	419	113	27.0%	1.024	0.763–1.376	0.872	7.1 (2 1 3)	4.8
	Regular sleep × Bedtime T3	243	61	25.1%	0.996	0.681–1.457	0.984	8.0 (1 1 1)	6.0
	Irregular sleep × Bedtime T1	39	16	41.0%	1.893	0.950–3.775	0.070	6.2 (16)	3.7
	Irregular sleep × Bedtime T2	22	8	36.4%	1.885	0.739–4.804	0.184	9.3 (9)	6.4
	Irregular sleep × Bedtime T3	59	14	23.7%	1.029	0.529–2.000	0.933	7.0 (25)	3.1

Female	Regular sleep × Bedtime T1	855	31	3.6%	ref			12.7 (4 4 4)	8.6
	Regular sleep × Bedtime T2	902	41	4.5%	1.411	0.858–2.320	0.175	13.5 (4 7 5)	8.7
	Regular sleep × Bedtime T3	526	30	5.7%	2.025	1.153–3.556	0.014	15.1 (2 5 2)	9.1
	Irregular sleep × Bedtime T1	34	6	17.6%	6.314	2.360–16.89	<0.001	20.8 (18)	18.2
	Irregular sleep × Bedtime T2	53	5	9.4%	3.222	1.168–8.887	0.024	20.9 (22)	15.3
	Irregular sleep × Bedtime T3	127	13	10.2%	3.827	1.839–7.964	<0.001	21.2 (65)	13.5
OR, odds ratio; CI, confidence interval									
Adjusted for age, sex, METs, sleep duration (T1-T3), drinking and smoking statuses, and the current use of medication for sleeping pills									

## 3 Results

Table 1 shows the baseline characteristics of participants according to sleep regularity or irregularity, including 3,546 and 334 participants in the sleep regular and irregular sleep groups, respectively. The average age of participants in the irregular sleep group was 55.5 years, which was younger than that in the regular sleep group (mean, 57.7 years). Sleep duration was associated with a significant reduction in the irregular sleep group compared with the regular sleep group. In addition, the bedtime of the irregular sleep group was significantly later than that of the regular sleep group.

Table 2 shows the relationship between MetS, irregular sleep, bedtime, and sleep duration. The irregularity of sleep was significantly associated with MetS (Model 3, OR 1.231, 95% CI 1.101–1.375; *p* < 0.001). On the contrary, the bedtime T1-3 group (T1 as the reference) and sleep duration T1-3 group (T1 as the reference) were not significantly associated with MetS. When analyzed separately according to sex, the irregular sleep group was predominantly associated with MetS in females, whereas none of the items were significantly associated with MetS in males.

Table 3 shows the interaction of MetS with sleep regularity and sleep duration, stratified by multiplying the two groups of sleep regularity/irregularity and the three groups of sleep duration T1-3. In the regular sleep group, there was no significant relationship with MetS, regardless of sleep duration. In contrast, in the sleep irregular group, there were significant relationships with MetS regardless of sleep duration (T1: OR 1.992, 95% CI 1.150–3.449; T2: OR 1.997, 95% CI 1.149–3.472; T3: OR 2.035, 95% CI 1.008–4.110). In addition, leptin levels were high in the irregular sleep group. Comparing the leptin values in the irregular sleep group, the results showed a U-shaped curve for sleep duration (T1: 17.7, T2: 14.0, and T3: 18.9). Leptin levels in females were markedly elevated in the irregular sleep group.

Table 4 shows the interaction of MetS with sleep regularity and bedtime, stratified by multiplying the two groups of sleep regularity/irregularity and the three groups of bedtime T1-3. As shown in Table 3, Table 4 shows that the regular sleep group was not significantly related to MetS, regardless of bedtime, whereas the irregular sleep group was significantly related to MetS (T1: OR 2.703, 95% CI 1.504–4.859; T2: OR 2.207, 95% CI 1.117–4.362; T3: OR 1.713, 95% CI 1.049–2.796). Leptin levels were higher in the irregular sleep group than in the regular sleep group and were also markedly elevated in females in the irregular sleep group.

## 4 Discussion

A number of systematic reviews and *meta*-analyses have shown a relationship between sleep and MetS (Krittanawong et al., 2019, Xi et al., 2014, Xie et al., 2021). In many of these studies, short and long sleep durations were associated with MetS. Several studies have also shown a U-shaped association between sleep duration and MetS (Ju and Choi, 2013, Krittanawong et al., 2019, Ohkuma et al., 2014, Smiley et al., 2019, Watanabe et al., 2018). However, this study showed that neither short sleep duration nor long sleep duration was associated with MetS. There are several possible reasons for this finding. The definitions of long and short sleep durations vary among papers. In this study, the groups were divided into tertile categories (T1: sleep duration <6 h, T2: 6–<7h, and T3: ≥7h). According to a recent *meta*-analysis, which concluded that cross-sectional studies showed a U-shaped association, the systematic review articles defined the short sleep group as those with sleep duration ≤5 h, <6h, and <7 h. In addition, the group with long sleep duration was defined as those with sleep duration ≥8 h, ≥9h, and ≥10 h, with discrepant definitions among studies (Xie et al., 2021). In general, sleep duration varies among cultures and ethnicities. Organisation for Economic Co-operation and Development statistics via the Gender Data Portal show that daily average sleep is 508 min in Britain, 513 min in France, 516 min in Spain, 528 min in the United States, and 542 min in China. Many countries also have an average of >500 min. On the other hand, Japan has the shortest average of 442 min (Organisation for Economic Co-operation and Development statistics via the Gender Data Portal, 2021). There are various causes of long and short sleep durations. It has been reported that depression, low socioeconomic status, undiagnosed medical disease, poor physical health, and low physical activity could result in long sleep duration (Krueger and Friedman, 2009, Patel et al., 2006). Young children, being unmarried, working long hours, and measures of poor health, including acute and chronic respiratory conditions, pain, and anxiety, increase the odds of short sleep duration (Krueger and Friedman, 2009). The causes were not completely adjusted. However, we investigated the interaction of MetS with sleep regularity and duration and found that an irregular sleep was significantly associated with MetS regardless of sleep duration. When analyzed separately according to sex, a similar trend was observed in females. There was no significant advantage in males. However, compared with the association between MetS and sleep duration and bedtime in males, there was an association with sleep irregularity. Therefore, we conclude that MetS is related to sleep irregularity rather than sleep duration.

Moreover, we examined the interaction of MetS with sleep regularity and bedtime, stratified by multiplying the two groups of sleep regularity/irregularity and the three groups of bedtime T1-3. This study showed that irregular sleep was associated with MetS regardless of bedtime. Notably, while the accuracy of bedtimes in the regular sleep group may be reasonable to some extent, the accuracy of bedtimes and sleep duration in the irregular sleep group may not be valid. This study examined the relationship between sleep irregularity and MetS and the relationship with bedtime was subsidiary data. We do not discuss this further in this study; however, we consider that further research is needed on the relationship between bedtime and MetS. In this study, we focused on sleep irregularity, which the participants assessed, and examined its relationship with MetS. The present study is the first to demonstrate the relationship between subjective sleep irregularity and MetS.

Circadian rhythm is an endogenous rhythm with a period of approximately 24 h that is entrainable and persists in the absence of external time cues (Potter et al., 2016). In modern society, shift work, artificial light at night, and reduced exposure to natural light during the day are becoming prevalent, which can easily cause disruptions in the circadian rhythm. As a result, it is likely that sleep tends to be irregular. According to a previous review, several common sleep problems, including insufficient sleep schedules, insomnia, sleep apnea, narcolepsy, shift work, and shift work disorder are known to cause sleep deficiencies that likely contribute to metabolic diseases (Depner et al., 2014). The relationship between circadian rhythm, metabolism, and sleep is complicated by a variety of factors. For example, it has been reported that there is a bidirectional relationship between metabolic dysfunction and sleep problems (Depner et al., 2014). Sleep irregularity, which is an exposure factor, can be considered to represent the deviation of the circadian rhythm, and it is considered appropriate to relate it to MetS. Leptin is produced predominantly by the adipose tissue and regulates energy homeostasis, neuroendocrine function, and metabolism (Friedman and Halaas, 1998, Kelesidis et al., 2010). In a healthy body, increased adipose depots increase leptin production and its circulating levels, which typically trigger a response to reduce feeding and promote energy expenditure (Cui et al., 2017). However, individuals with obesity might have reduced sensitivity or a failure of response of the brain to leptin, showing a decrease in the ability of leptin to suppress appetite or enhance energy expenditure, which causes an increased food intake and finally leads to weight gain, obesity, and cardiovascular diseases (Liu et al., 2018). Leptin levels is correlated with the percentage of body fat, and most obese individuals are insensitive to endogenous leptin production, suggesting leptin resistance (Considine et al., 1996). Some sex-based differences in leptin levels were observed. Leptin levels increased three times more rapidly with progressive obesity in females, as measured either according to BMI or percentage body fat. A further difference is that induced hyperinsulinemia led to an increase in leptin levels in females, but not males (Kennedy et al., 1997). We measured the leptin level in approximately half of the participants in this study. Leptin levels were significantly elevated in the irregular sleep group in females, regardless of sleep duration and bedtime. These results suggest that sleep irregularity, rather than sleep duration or bedtime, is associated with obesity and leptin resistance, or it is probable that leptin resistance has already been acquired in patients with MetS. In addition to sleep duration and bedtime, this indicates that the relationship between sleep irregularity and MetS is plausible.

This study is novel because few studies have examined the relationship between sleep irregularity and MetS from the perspective of participant subjectivity. However, several limitations of this study need to be acknowledged. First, this study had a cross-sectional design, and we were not able to observe the participants in the time series. The causes and effects could be sufficiently demonstrated in this study alone; therefore, further research in a chronological order is needed. Second, this study used subjective questionnaires to acquire about sleep irregularity, duration, and bedtime that may not accurately reflect the actual daily sleep. In this study, participants were asked “Do you have regular hours in which to sleep and wake up?” (regular or irregular) to obtain information concerning sleep irregularity. Therefore, “irregular” included factors that made sleep irregular, such as night work and sleep interruption. The validity of these sleep items, which were acquired using subjective questionnaires, has not been established; however, we consider that their versatility, including their many meanings, makes them suitable for application in future epidemiological studies. Third, participants were limited to a Japanese population. As previously mentioned, according to the OECD survey, while many countries around the world have exceeded 500 min of average sleep, Japanese people have the shortest average sleep duration (442 min), making the Japanese population a unique group. It remains unclear whether similar results would be observed in different ethnic groups or among other cultures. Fourth, in this study, we did not have information on the types of jobs that were significantly related to social jetlag.

## 5 Conclusions

Although many studies have shown a link between sleep and MetS, especially in terms of sleep duration, this study showed that irregular sleep is more strongly associated with MetS than sleep duration or bedtime. Regular sleep may be important to prevent MetS.

## Funding

This study was supported by Grants-in-Aid for Scientific Research for Priority Areas of Cancer (No. 17015018) and Innovative Areas (No. 221S0001) and by the Japan Society for the Promotion of Science (JSPS) KAKENHI Grant (No. 16H06277 [CoBiA]) from the Japanese Ministry of Education, Culture, Sports, Science and Technology.

### CRediT authorship contribution statement

**Yasuhiro Ogura:** Data curation, Formal analysis, Methodology, Project administration, Writing – original draft. **Teruhide Koyama:** Conceptualization, Data curation, Funding acquisition, Investigation, Methodology, Project administration, Validation, Writing – original draft. **Etsuko Ozaki:** Data curation, Investigation, Project administration, Writing – review & editing. **Chie Omichi:** Data curation, Supervision, Writing – review & editing. **Ritei Uehara:** Funding acquisition, Investigation, Methodology, Project administration, Supervision, Writing – review & editing.

## Declaration of Competing Interest

The authors declare that they have no known competing financial interests or personal relationships that could have appeared to influence the work reported in this paper.
